# Synergistic antioxidant and antibacterial effects of a Zn-ascorbate metal–organic framework loaded with marjoram essential oil†

**DOI:** 10.1039/d4na00519h

**Published:** 2024-07-17

**Authors:** Rana R. Haikal, Noha El Salakawy, Alaa Ibrahim, Shaimaa L. Ali, Wael Mamdouh

**Affiliations:** a Department of Chemistry, School of Sciences and Engineering, The American University in Cairo (AUC) AUC Avenue, P. O. Box 74 New Cairo 11835 Egypt wael_mamdouh@aucegypt.edu

## Abstract

Antimicrobial resistance (AMR) has become an immense threat to public health leading to an urgent need for development of new technologies to tackle such a challenge. Plant-based drugs, specifically essential oils (EOs) and plant extracts, have shown significant potential as effective green antimicrobial agents. However, they suffer from high volatility and low thermal stability resulting in their inefficient utilization in commercial settings. Among the various nanoencapsulation technologies reported, metal–organic frameworks (MOFs) have been recently investigated as potential nanocarriers of EOs in attempt to enhance their stability. Herein, we report the utilization of Zn-ascorbate MOF for the encapsulation of marjoram essential oil (MEO) with synergistic antioxidant and antibacterial activities. The prepared composite was thoroughly characterized *via* a number of techniques and its antibacterial performance was investigated against various strains of Gram-negative and Gram-positive bacteria. The results demonstrated that the antioxidant activity originated from the ascorbic acid ligand (l-Asc), while the antibacterial activity originated from Zn^2+^ ions as well as encapsulated MEO.

## Abstract 2

Marjoram essential oil was successfully loaded onto Zn-ascorbate MOF. The antioxidant activity of the composite originated from the l-ascorbic acid ligand, while the antibacterial activity bacteria demonstrated the synergistic activity of Zn^2+^ ions and encapsulated oil.

## Graphical abstract

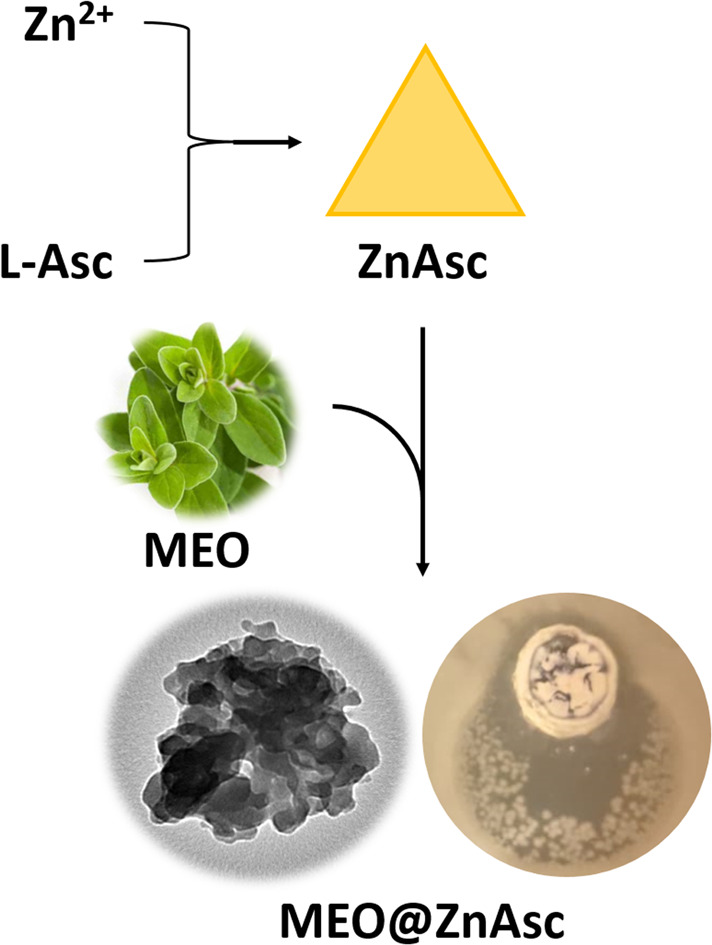

## Introduction

Antimicrobial resistance (AMR) has been declared by the World Health Organization (WHO) as one of the top 10 global public health threats in 2019, which escalated to be one of the top 3 priority health threats as identified by the European Commission (Health Emergency Preparedness and Response Authority, HERA) and the member states in July 2022. By 2050, the mortality rate related to AMR is estimated to increase to up to 10 million deaths and the world economy would be financially burdened up to USD 100 trillion forcing 28 million people into extreme poverty, 93% of whom are in developing countries. Therefore, there is an urgent need for development of novel antimicrobial agents and technologies to combat this threat.

Unfortunately, the development of new antimicrobial agents is very costly and not speedy enough to surpass the advancement of AMR; thus, novel strategies are being explored to enhance the efficacy of current antimicrobials as well as search for alternative solutions. Among the various methods currently investigated, including using antimicrobial peptides, bacteriophages, and metal nanoparticles, using natural products remains the safest and least complex approach.^1,2^ Recently, research on plant-based drugs has expanded and they have been demonstrated to show promising activity against several drug-resistant microbes.^3^ Specifically, essential oils (EOs) and plant extracts have demonstrated a broad spectrum of antimicrobial activity against human pathogens.^4,5^ Some reports have also confirmed the potential use of EOs as alternatives to antibiotics in animal production due to their antimicrobial activity against foodborne pathogens.^6,7^ Such remarkable performance can be owed to the different mechanisms of action as a result of the multicomponent system of EOs and plant extracts, which explains the enhanced efficacy of existing antimicrobials.^8^ One example, marjoram EO (MEO), scientifically known as *Origanum majorana*, has been found to possess significant antimicrobial activity against a wide range of microorganisms. It has long been reported to exhibit efficacy against both bacteria and fungi, making it a versatile nutraceutical compound that can be used as an effective and safe antimicrobial agent.^9–12^ Moreover, researchers demonstrated that combination of MEO increased the susceptibility of multidrug-resistant bacteria to existing antibiotics through disrupting the lipid bilayer membrane subsequently improving the antibiotics' permeability.^8,13^ The high content of oxygenated terpenes in MEO was also reported to inhibit microbial oxygen uptake and oxidative phosphorylation, thereby synergistically contributing to the antimicrobial activity.^14^

Despite the beneficial properties of EOs, their volatility and low thermal stability are challenging factors that restrict their utilization in commercial settings. Micro- and nanoencapsulation have demonstrated great promise as strategies to enhance the stability and control the release of encapsulated EOs in various applications.^15–18^ While there are many physical and chemical methods of encapsulating EOs reported, some limitations still exist. Specifically, the majority of chemical methods, including using polymeric nanoparticles, liposomes, and nanoemulsions, suffer from relatively low thermal, chemical, and colloidal stabilities.^16^ Therefore, researchers have recently investigated metal–organic frameworks (MOFs) and their nanocomposites for their inherent antimicrobial properties or carriers for antimicrobial agents in different biomedical and food packaging applications.^19,20^ MOFs are crystalline microporous 3D networks of metal ions/clusters interconnected *via* multitopic organic ligands. Their unique properties, such as high surface area, tuneable porosity and functionality, and good physical and chemical stability, have attracted significant attention in many industrial, environmental, and biomedical applications.^21,22^ Considering the hybrid inorganic–organic composition of MOFs, they have also shown significant antimicrobial properties by acting as reservoirs for bactericidal metal ions (*e.g.* Cu^2+^, Ag^+^, Zn^2+^, *etc.*) and/or bactericidal linkers that are released gradually due to slow degradation.^23,24^ Therefore, MOFs are ideal candidates for encapsulation of EOs in an attempt to enhance their thermal stability and control their release in a sustained manner. Indeed, several examples of MOFs have been investigated towards the encapsulation of EOs, plant extracts, and natural compounds for a variety of biomedical applications. In specific, cyclodextrin-based MOFs have been heavily reported as carriers used in the encapsulation of thymol,^25,26^ clove EO,^27^ curcumin,^28^ olivetol,^29^*Origanum compactum* EO,^30^ lavender EO,^31^ and reseveratrol,^32^ among others. Other MOFs have also been reported, where the effect of loading Cu-BTC with *Nigella sativa* EO and wheat germ EO on chronic murine taxoplasmosis was investigated.^33^ The antimicrobial properties of thymol-loaded zinc-based MOFs were also explored.^34^ Additionally, the antimicrobial activity of carvacrol was prolonged *via* loading into the intrinsically redox-active MIL-100(Fe).^35^

Herein, we demonstrate an edible biocompatible MOF based on zinc and l-ascorbic acid, which was post-synthetically loaded with MEO *via* a simple wet incipient impregnation method. This MOF was judiciously selected owing to the widely reported potent antimicrobial activity of zinc ions and antioxidant activity of ascorbic acid.^36–38^ The MEO@ZnAsc composite was then thoroughly characterized and its antioxidant as well as antibacterial performances were tested. The synergy between the antioxidant activity of ascorbic acid and the antibacterial activities of Zn^2+^ and MEO was evident, which verifies the developed composite to be a promising green and sustainable solution for AMR.

## Results and discussion

### Synthesis and characterization of MEO@ZnAsc

**Fig. 1:**
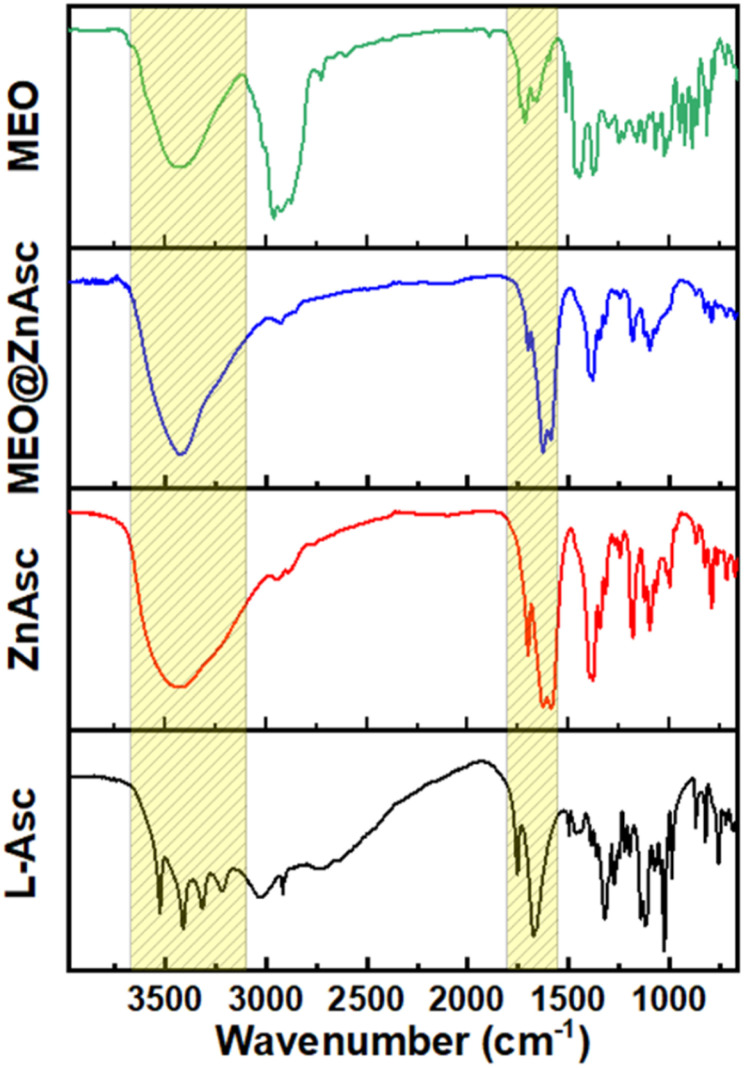
FT-IR spectra of l-Asc, ZnAsc, MEO@ZnAsc, and MEO.

The chemical composition of the commercial MEO was first analysed by gas chromatography coupled with mass spectrometry (GC-MS), Table S1,† which demonstrated the major components to be terpinene-4-ol (32.72%), *o*-cymene (15%), γ-terpinene (7.82%), α-terpineol (4.49%), α-terpinene (4.38%), sabinene (3.2%), and d-limonene (3.18%). Such a composition is consistent with those reported in the literature for commercial oil as well as various extracts.^39–41^ Zinc ascorbate-based MOF (ZnAsc MOF) was then prepared according to a modified solvothermal procedure recently reported by Tajnšek *et al.*^42^ Loading of MEO within ZnAsc was performed post-synthetically *via* wet incipient impregnation in ethanol at room temperature (see the Experimental section for further information). The pristine MOF (ZnAsc MOF) and the MEO-loaded composite (MEO@ZnAsc) were characterized by a number of techniques to validate the successful encapsulation of MEO as well as to investigate the structural stability of the composite. Fig. 1 demonstrates the Fourier transform infrared (FT-IR) spectra of the l-ascorbic acid precursor (l-Asc), ZnAsc, MEO@ZnAsc, and MEO. It is evident that the four rather sharp O–H stretching vibration frequencies of l-Asc (3526, 3410, 3316, and 3217 cm^−1^)^43^ transform into a single broad O–H stretching centered at 3434 cm^−1^ in ZnAsc. This confirms the successful deprotonation and coordination of all hydroxyl groups of l-Asc with Zn(ii) centers within the ZnAsc framework. The singular and broad nature of the observed O–H stretching in ZnAsc can be attributed to hydrogen bonding of the independent hydroxyl group with residual solvent molecules, which correlates well with the previously reported chemical formula Zn_3_(Asc)(OH).^42^ Additionally, the carbonyl stretching frequency (*ν*_C=O_) of the lactone ring shifted from 1754 cm^−1^ in l-Asc to 1702 cm^−1^ in ZnAsc, which further validates the electron withdrawing effect of coordination to Zn(ii) centers. Another significant change is the shift and splitting of the C=C stretching vibration frequency of l-Asc (1674 cm^−1^) to lower wavenumbers in ZnAsc (1626 and 1586 cm^−1^). By examining the FT-IR spectrum of MEO@ZnAsc, it is mostly similar to that of ZnAsc with some minimal changes indicating the structural stability of ZnAsc during the loading process of MEO. The shift of the O–H and carbonyl stretching frequencies to lower wavenumbers (3415 and 1700 cm^−1^, respectively) suggests the effective interaction of MEO with the ZnAsc framework *via* hydrogen bonding.

The absence of apparent peaks characteristic of MEO in the FTIR spectrum of MEO@ZnAsc is most likely due to the relatively low loading capacity of MEO (5.5 wt%) within the composite as demonstrated *vide infra*.

**Fig. 2:**
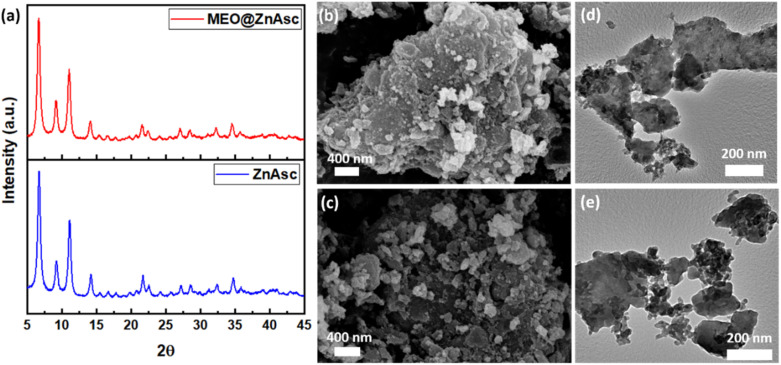
(a) XRD patterns of ZnAsc and MEO@ZnAsc, and (b and c) SEM and (d and e) HRTEM images of ZnAsc and MEO@ZnAsc, respectively.

Furthermore, the crystallinity of the prepared MOF and composite was assessed by X-ray powder diffraction (XRD), where the patterns are shown in Fig. 2a. ZnAsc revealed characteristic peaks similar to those in previous reports,^42^ which are preserved in that of MEO@ZnAsc indicating maintained structural integrity and crystallinity following loading of MEO. Moreover, the morphology and particle size of the pristine MOF and composite were analysed by scanning electron microscopy (SEM) and transmission electron microscopy (TEM), the images of which are shown in Fig. 2b–e. Significant aggregation of smaller nanoparticles is evident, which is consistent with the near-zero surface charge.^42^ Table S2† demonstrates the average crystallite/particle size to range between 16 and 23 nm *via* different calculation methods. It should be noted that the nanoparticle size did not change significantly after MEO loading further confirming the intact structural integrity of ZnAsc. To further validate the encapsulation of MEO within the ZnAsc framework, N_2_ sorption isotherms were measured (Fig. S1†) from which the Brunauer–Emmett–Teller (BET) surface areas, pore volumes, and pore size distributions were calculated (Table 1 and Fig. S1†). Both samples demonstrate a type I(b)-like isotherm with an evidently sharp rise in the adsorbed amount near saturation pressures, which signifies the presence of a complex interparticulate pore network consistent with the substantial aggregation observed in SEM and TEM images.^44^ Such aggregation of smaller nanoparticles leads to the creation of a hierarchical structure with mixed micro-/mesopores, where the pore size distribution revealed two sets of micropores at 1.4 and 1.6 nm and a broader range of mesopores in the range 6–30 nm (Fig. S1†). The overall reduction in BET surface area and pore volumes of MEO@ZnAsc in comparison to ZnAsc confirmed the successful entrapment of MEO within the pores of ZnAsc.

**Table 1:** BET surface areas and pore volumes of ZnAsc and MEO@ZnAsc calculated from N_2_ sorption isotherms

Parameter	ZnAsc	MEO@ZnAsc
BET surface area (m^2^ g^−1^)	400	298
Total pore volume (cc g^−1^)	0.522	0.369
Micropore volume (cc g^−1^)	0.134	0.099

**Fig. 3:**
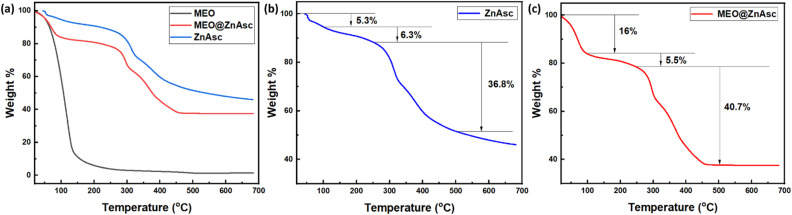
(a) TGA profiles of MEO, ZnAsc and MEO@ZnAsc with detailed degradation steps of ZnAsc (b) and MEO@ZnAsc (c), respectively.

Additionally, the thermal stabilities of ZnAsc and MEO@ZnAsc were investigated using thermogravimetric analysis (TGA). As expected of a volatile essential oil, MEO has a weak thermal stability and lost ∼85% of its weight before 120 °C (Fig. 3a).^45^ Further examination of the TGA profile of ZnAsc (Fig. 3b) confirms the obtained structure of Zn_3_(Asc)(OH). The weight loss before 100 °C is attributed to residual solvent molecules, which correspond to 5.3 wt%. The additional slight weight loss from 100 °C to 250 °C corresponding to 6.3 wt% can be ascribed to the independent hydroxyl group.

Thermal degradation of ZnAsc effectively starts at 250 °C in a two-step process corresponding to a total weight loss of 36.8 wt% between 250 °C and 500 °C. This major weight loss would be due to thermal degradation of the ascorbic acid ligand, where the first step is most likely dehydroxylation followed by carbonization of the carbon backbone.^46^ The residual solid remaining corresponding to ∼48 wt% is most likely thermally stable ZnO. Overall, the changes in the O–H stretching band structure as well as the shifts in the C=O and C=C stretching frequencies from the FTIR spectra correlate well with the chemical formula Zn_3_(Asc)(OH) attained from the TGA data analysis. Similarly, closer inspection of the TGA profile of the MEO@ZnAsc composite (Fig. 3c) reveals significant differences. First, the weight loss below 100 °C is significantly higher (16 wt%), indicating incomplete dryness of the composite as would be expected from drying at rt (see the Experimental section for more details). While previous reports ascribed such weight loss to evaporation of entrapped/adsorbed oil,^47^ considering that MEO starts evaporating in the same temperature range, it is more likely to be due to residual solvent in this case. This is because the composite was washed with ethanol to remove additional adsorbed MEO, which was further confirmed by the absence of apparent characteristic peaks in the FTIR spectrum of MEO@ZnAsc (*vide supra*). Nevertheless, a similar weight loss of 5.5 wt% between 100 °C and 250 °C was observed and the major degradation with a total weight loss of 40.7 wt% occurred between 250 °C and 500 °C. However, an additional third step becomes more discernible at around 400 °C as evident from the first derivative of the TGA profile of MEO@ZnAsc (Fig. S2†). This is most likely due to degradation and/or carbonization of entrapped MEO in the composite, which further confirms that the MEO is well-encapsulated within the pores of the MOF rather than being adsorbed on the surface. By calculating the difference between the weight loss of ZnAsc and MEO@ZnAsc in the temperature range of 250–500 °C, it can be concluded that the amount of MEO entrapped is ∼4 wt%. Such a loading amount is comparable to that in other reports for utilization of MOFs for essential oil encapsulation, where loading capacity% values in the range of 3.96–5.8% were reported.^29,32,34^ Nevertheless, a significantly higher carvacrol content of 26.7 wt% was attained by loading within MIL-100(Fe) NPs,^35^ which is most likely due to the large BET surface area of MIL-100(Fe) (1491 m^2^ g^−1^). This shows the substantial structure–property relationship between the carrier MOF structure and subsequent loading capacity of entrapped oil, which can be notable for further investigations.

### Antioxidant activity

**Fig. 4:**
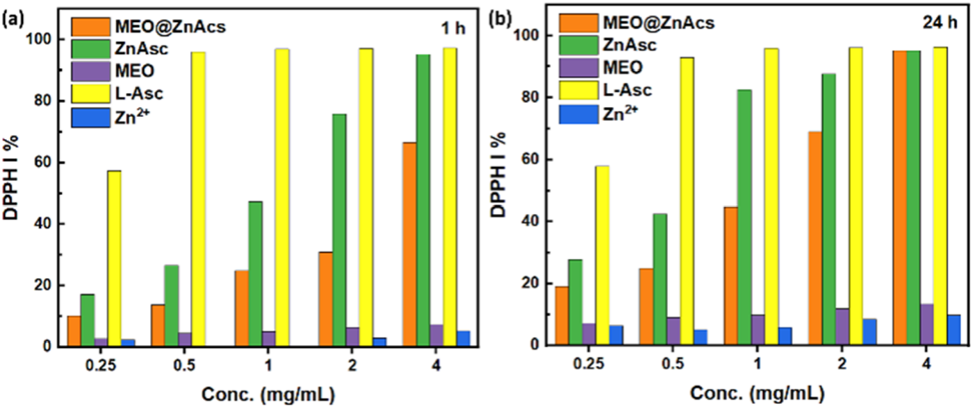
DPPH I% values of MEO@ZnAsc and equivalent concentrations of ZnAsc, MEO, l-Asc, and Zn^2+^ after (a) 1 h and (b) 24 h.

The antioxidant activity of the developed MEO@ZnAsc composite was assessed using 2,2-diphenyl-1-picrylhydrazyl (DPPH) radical scavenging assay. The same procedure was applied to ZnAsc, MEO, l-Asc, and zinc acetate dihydrate (Zn^2+^) for comparison using equivalent weights of the corresponding amounts present in MEO@ZnAsc based on the TGA data analysis. As evident in Fig. 4a, MEO@ZnAsc, ZnAsc, and l-Asc demonstrated dose-dependent radical-scavenging activities after 1 h of incubation, while MEO and Zn^2+^ ions demonstrated a relatively minimal response. This reveals that l-Asc is the major contributing component towards the composite's antioxidant activity. It is also evident that l-Asc achieves the maximal response (>95%) at an equivalent dose of 0.5 mg mL^−1^ after 1 h, compared to a dose of 4 mg mL^−1^ for ZnAsc. Such observations can be explained by the fact that the active antioxidant constituent (l-Asc) is tightly bound within the MOF and it is not readily available in solution for DPPH radical scavenging. Hence, the gradual collapse of ZnAsc in solution releases l-Asc to show its antioxidant activity, which is still quite appreciable. This is further confirmed by the increase in DPPH inhibition% (DPPH I%) values of ZnAsc after 24 h of incubation (Fig. 4b), where they increased by 60–75% at low doses (0.25–1 mg mL^−1^) compared to the invariable response of l-Asc in the same time frame. Therefore, the assimilation of l-Asc within the stable framework of ZnAsc MOF allowed for its sustained release and maintaining adequate antioxidant activity over an extended period of time. Compared to the antioxidant activity of ZnAsc, the oil-loaded MEO@ZnAsc composite demonstrated overall lower DPPH I% values after both 1 and 24 h of incubation. This observation suggests that the encapsulation of MEO retarded the release of l-Asc from MEO@ZnAsc or, in other words, stabilized the MOF structural integrity, which can be explained by the strong interaction between MEO and ZnAsc as reflected by other aforementioned characterization techniques (FTIR, XRD, SEM and TGA). Similarly, the DPPH radical-scavenging activity of MEO@ZnAsc increased after 24 h of incubation by an average of 83% over the whole dose range.

### Antimicrobial activity

The antimicrobial activities of pristine MOF as well as MEO-loaded MOF were assessed qualitatively by measuring the inhibition zone diameters using the agar well diffusion method against several Gram-negative and Gram-positive bacterial strains (Table 2). The results showed that the antibacterial activity of the composite, MEO@ZnAsc, is significantly higher than that of pristine ZnAsc across all bacterial strains tested confirming the enhanced performance of the MOF post loading with MEO. To further investigate the activity of the individual components, equivalent concentrations of MEO, l-Asc, and Zn^2+^ were tested under the same conditions. The observed inhibition zones clearly validate that the major antibacterial activity originated from MEO and Zn^2+^, which is widely reported in the literature.^10,36^ Several examples of Zn-based MOFs have been previously investigated regarding their antimicrobial performance against *Escherichia coli* (*E. coli*) and *Staphylococcus aureus* (*S. aureus*) as model strains for Gram-negative and Gram-positive bacteria, respectively (Table S3†). It is worth noting that the antibacterial performance of pristine ZnAsc MOF is somewhat lower than that of most reported Zn-based MOFs despite the higher Zn wt% (Table S3†). This could be explained by the fact that inhibition zone diameters in agar well diffusion methods depend on multiple factors other than the % Zn composition, which include the MOF stability, rate/amount of Zn^2+^ released, and concentration/volume of the MOF suspension added to the well. The smaller inhibition diameters of ZnAsc can merely be ascribed to the slower degradation rate due to higher stability of the Zn cluster, thereby lowering the release rate of Zn^2+^ into the agar medium. This was also exhibited by MOF-5 showing lower antibacterial activity due to the higher stability of the Zn acetate cluster compared to the monometallic Zn node in TMU-3 despite the similar Zn wt% in both MOFs.^48^ As for the amount of Zn^2+^ released from ZnAsc, it has been reported to largely depend on the solvent medium.^42^

**Table 2:** Inhibition zones against the different bacterial strains using the agar well diffusion method

Bacterial strain	Inhibition zone diameter (mm ± SD)				
	MEO@ZnAsc	ZnAsc	MEO	l-Asc	Zn^2+^
**Gram negative bacteria**					
*Escherichia coli*	13.83 ± 0.2	10.33 ± 0.5	8.33 ± 0.5	0	9 ± 0
*Salmonella typhi*	9.5 ± 0.5	8 ± 0.5	8.5 ± 0.5	0	11 ± 0
*Pseudomonas putida*	7 ± 0	4 ± 0	8 ± 0	0	9 ± 0

**Gram positive bacteria**					
*Staphylococcus aureus*	21.9 ± 0.1	8 ± 0	8.66 ± 0.5	1.75 ± 0.3	11 ± 0
MRSA	12 ± 0	11.5 ± 0.7	7.25 ± 0.3	7.5 ± 0.7	11.75 ± 0.7

To further validate the enhanced antibacterial performance of the prepared oil-loaded composite, the IC_50_ values of MEO@ZnAsc, ZnAsc, MEO, and Zn^2+^ against *E. coli* and *S. aureus* as model strains were measured using the broth microdilution method (Table 3 and Fig. S3†). The results showed that the composite, MEO@ZnAsc, obtained the lowest IC_50_ values indicating its potency owing to the multiple mechanisms of action of its individual components. Nevertheless, the IC_50_ values for ZnAsc are comparable to those reported for other MOFs.^49^ The only surprising finding was that the IC_50_ values for ZnAsc and MEO@ZnAsc against *S. aureus* are almost identical despite the large difference in inhibition zone diameters. This can be ascribed to the better release and diffusion through agar of MEO from MEO@ZnAsc than Zn^2+^ from ZnAsc. It is also worth noting that the concentrations used in the agar well diffusion methods are equivalent to the composition of the individual components in MEO@ZnAsc as demonstrated in the TGA profile, while in the broth microdilution method, the same concentration range was utilized for all samples tested. Overall, both inhibition zone diameters and IC_50_ values confirm the enhanced antibacterial activity of the composite compared to the separate components, which further validates the synergistic activity due to multiple mechanisms of action.

**Table 3:** IC_50_ values against *E. coli* and *S. aureus* according to the broth microdilution method

Sample	IC_50_ (μg mL^−1^)	
	*E. coli*	*S. aureus*
MEO@ZnAsc	112	238
ZnAsc	156	233
MEO	161	362
Zn^2+^	218	435

## Experimental

### Materials and methods

Zinc acetate dihydrate (98%) and l-ascorbic acid (l-Asc) were purchased from Loba Chemie and Research Lab Fine Chem Industries, India, respectively. Ethanol (99%) and the 2,2-diphenyl-1-picrylhydrazyl radical (DPPH) were purchased from Sigma-Aldrich. Marjoram essential oil was obtained from the Areej company, Egypt. DPPH absorbance values were measured using an Agilent Cary 3500 compact UV-Vis spectrophotometer. Thermogravimetric analysis (TGA) was performed using a TA Instruments Q50. Fourier-transform Infrared spectra were obtained using a Nicolet 380, ThermoScientific. X-ray powder diffraction patterns were acquired using a D8 Discover, Bruker Advanced X-ray solutions. Data acquisition was carried out using Cu K_α_ radiation and a Ni filter. The X-ray tube was operating at 40 kV and 30 mA current. Scanning electron microscopy images were obtained using a Leo Supra 55 with an in-lens detector.

N_2_ sorption isotherms were measured using a Micromeritics ASAP2020 after degassing the samples at 140 °C for 6 h. The Brunauer–Emmett–Teller (BET) surface areas were determined from the nitrogen adsorption isotherms collected at 77 K. Electron microscopy imaging was performed using an FESEM, Leo Supra 55-Zeiss Inc., Germany, and a Tecnai G2 F20 U-Twin for SEM and HRTEM, respectively. GC-MS analysis was performed on an Agilent 7890B gas chromatogram interfaced with a mass-selective detector (MSD, Agilent 7000 Triple Quad) equipped with an apolar Agilent Hp-5ms (5%-phenylmethylpolysiloxane) capillary column (30 m × 0.25 mm i. d. × 0.25 μm film thickness). The carrier gas was helium with a linear velocity of 1 mL min^−1^ The injector and detector temperatures were 280 °C. Injection mode, split; split ratio 1 : 10; volume injected 1 μL of the sample. The gradient temperature program was set as follows: the initial oven temperature was set at 40 °C for 1 min, then was elevated to 150 °C at a rate of 4 °C min^−1^ and retained at 150 °C for 6 min, and finally was elevated at a rate of 4 °C min^−1^ to 210 °C and kept at this temperature for 1 min. The MS operating parameters were as follows: ionization potential 70 eV, interface temperature 220 °C, and acquisition mass range 50–450. The identification of components was based on a comparison of their mass spectra and retention times by computer matching with the NIST and WILEY library as well as comparison of the fragmentation pattern of the mass spectra with those reported in the literature.

### Synthesis of zinc ascorbate (ZnAsc) MOF

In a glass vial, zinc acetate dihydrate (1.5 g, 6.8 mmol) and l-ascorbic acid (l-Asc) (600 mg, 3.4 mmol) were dissolved in ethanol (20 mL). The solution was then stirred at 500 rpm at 120 °C overnight. After cooling to rt, the suspension was centrifuged at 7000 rpm for 5 min. The supernatant was discarded and the solid was washed with ethanol several times to remove the excess unreacted precursors. The solid was then dried in an oven at 90 °C for further analysis.

### Synthesis of marjoram essential oil-loaded ZnAsc (MEO@ZnAsc)

Loading of MEO into ZnAsc was performed in ethanol at rt, where 150 mg of MEO was weighed and dissolved in 15 mL ethanol. 150 mg of ZnAsc was then weighed and added to the MEO ethanol solution and the obtained solution was stirred for 2 h at 250 rpm at rt. The suspension was then centrifuged at 7000 rpm for 5 min and washed once with ethanol and then left to dry in air at rt.

### Antioxidant activity

The antioxidant activity of the MEO and the developed ZnAsc and MEO@ZnAsc were investigated using 2,2-diphenyl-1-picrylhydrazyl (DPPH) free radical scavenging assay. Serial concentrations of MEO@ZnAsc ethanol suspension (0.25, 0.5, 1, 2, and 4 mg mL^−1^) were prepared. Similar dilutions of ZnAsc, MEO, l-Asc, and Zn^2+^ were also prepared using equivalent weights of MEO@ZnAsc. DPPH stock solution (4 mg in 100 mL) was prepared and kept in the dark. To 750 μL of sample solution, 3 mL of DPPH stock solution was added and the mixture was left in the dark for 24 h. For initial DPPH concentration measurement, a blank solution was prepared by adding 750 μL of pure ethanol instead of sample solution. After the specified amount of time, the suspensions were centrifuged and the absorbance of the remaining DPPH in the supernatants was measured using UV-Vis spectrophotometry at *λ* = 517 nm after incubation for 1 h and 24 h. The DPPH radical-scavenging activities (DPPH I %) were calculated as follows:
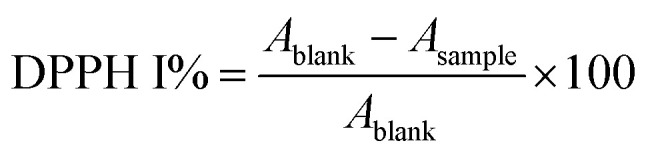


### Qualitative antimicrobial activity (agar well diffusion method)

The antimicrobial efficacy of the samples was assessed using the agar well diffusion method.^50^ In brief, Mueller–Hinton agar plates were prepared and then inoculated with 200 μL (OD 0.5 equivalent to 10^8^ CFU mL^−1^) of each of the tested bacterial strains. After that, a 6 mm hole was punched aseptically using a sterilized well cutter, where 100 μL of each sample (equivalent concentrations of 10 mg mL^−1^ of MEO@ZnAsc suspended in EtOH) was loaded in each well. Finally, the agar plates were incubated at 37 °C for 24 hours. All experiments were performed in triplicate, and the diameter of the inhibitory zone was determined as the mean ± standard deviation (SD).

### Quantitative antimicrobial activity (broth microdilution method)

The 50% inhibitory concentrations (IC_50_) of the samples against Gram-negative *E. coli* and Gram-positive *S. aureus* were analyzed using the broth microdilution method. Briefly, a serial dilution of the samples was added to 10^6^ CFU mL^−1^ of each bacterial strain in 96-well plates using Luria-Bertani (LB) broth medium in the concentration range of 0 to 10 mg mL^−1^. The plates were incubated at 37 °C for 20 hours, after which the optical density (OD_600_) was measured using a Tecan Infinite 200 PRO M Plex multimode microplate reader 30050303.

## Conclusions

Herein, we demonstrate the utilization of biocompatible Zn-ascorbate MOF (ZnAsc) as a nanocarrier for marjoram essential oil (MEO). The loading capacity was determined to be ∼4 wt% according to thermogravimetric analysis. The antioxidant activity of the prepared composite was analysed in comparison to its individual components, which demonstrated the activity to originate from the l-ascorbic acid ligand and in a sustained manner. The antibacterial activity was also investigated against several Gram-negative and Gram-positive bacterial strains, which demonstrated the synergistic activity of Zn^2+^ ions and encapsulated MEO. Despite the enhanced performance of MEO@ZnAsc relative to ZnAsc, further investigation of the structure–activity relationship regarding the stability of the Zn node/cluster and its antibacterial activity is essential. Moreover, the understanding of the effect of MOF topology on the loading capacity of encapsulated essential oils and plant extracts is still lacking. Nevertheless, the reported work demonstrates great potential of MOFs for the encapsulation of essential oils with antimicrobial properties in an attempt to develop sustainable solutions to AMR.

## Data availability

Data for this article are not publicly available, but could be shared upon request.

## Author contributions

RH contributed to conceptualization, methodology, investigation, and writing. NE, AI, and SL contributed to methodology, investigation, and review. WM contributed to supervision, review, and editing.

## Conflicts of interest

There are no conflicts to declare.

## Supplementary Material

NA-006-D4NA00519H-s001
