# Supplementary material for: Synergistic antioxidant and antibacterial effects of a Zn-ascorbate metal–organic framework loaded with marjoram essential oil
Source: Nanoscale Adv. 2024 Jul 17;6(18):4664–71. doi: 10.1039/d4na00519h (PMC11386125; doi:10.1039/d4na00519h)
Supplement: NA-006-D4NA00519H-s001 [file NA-006-D4NA00519H-s001.pdf]

Electronic Supplementary Material (ESI) for Nanoscale Advances.
This journal is © The Royal Society of Chemistry 2024

Supplementary Information

Synergistic antioxidant and antibacterial effect of Zn-ascorbate metal-organic framework loaded with Marjoram essential oil

*Rana R. Haikal, Noha El Salakawy, Alaa Ibrahim, Shaimaa L. Ali, and Wael Mamdouh**

Department of Chemistry, School of Sciences and Engineering, The American University in Cairo (AUC), AUC Avenue, P.O. Box 74, New Cairo 11835, Egypt

Table S1. Composition of marjoram essential oil (MEO) as purchased (analyzed by GC-MS).

No.	Compound	t_R (min)	% Peak area
1	Thujene	8.11	1.27
2	α-Pinene	8.31	0.88
3	Camphene	8.79	0.04
4	**Sabinene**	**9.67**	**3.20**
5	2-β-Pinene	9.75	0.43
6	β-Myrcene	10.32	0.89
7	**α-Terpinene**	**11.19**	**4.38**
8	***o*-Cymene**	**11.52**	**15.00**
9	**D-Limonene**	**11.63**	**3.18**
10	Eucalyptol	11.70	0.21
11	**γ-Terpinene**	**12.74**	**7.82**
12	*m*-Cresol	13.58	0.12
13	α-Terpinolene	13.78	2.42
14	*cis*-Sabinene hydrate	14.13	2.28
15	Linalool	14.24	1.37
16	*cis*-*p*-Menth-2-en-1-ol	14.97	0.83
17	1-Terpineol	15.64	0.75
18	**Terpinene-4-ol**	**17.14**	**32.72**
19	*p*-Cymen-8-ol	17.32	0.91
20	**α-Terpineol**	**17.51**	**4.49**
21	Isopulegol	17.65	0.48
22	*trans*-*p*-Menth-1-en-3-ol	18.07	0.62
23	Linalyl anthranilate	19.76	2.64
24	1,8-Cineole	21.08	0.32
25	Carvacrol	21.36	0.44
26	4,4-Dimehtylpent-2-enal	21.92	0.58
27	*cis*-Carveol	23.52	0.15
28	Caryophyllene	25.12	2.54
29	(+)-Aromadendrene	25.72	0.11
30	1,4-Dihydroxy-*p*-menth-2-ene	26.95	0.47
31	Ledene	27.46	0.34
32	(+)-Spathulenol	30.03	0.67

**33**	(-)-Caryophyllene oxide	30.21	0.91
	**Total identified**		**93.46**

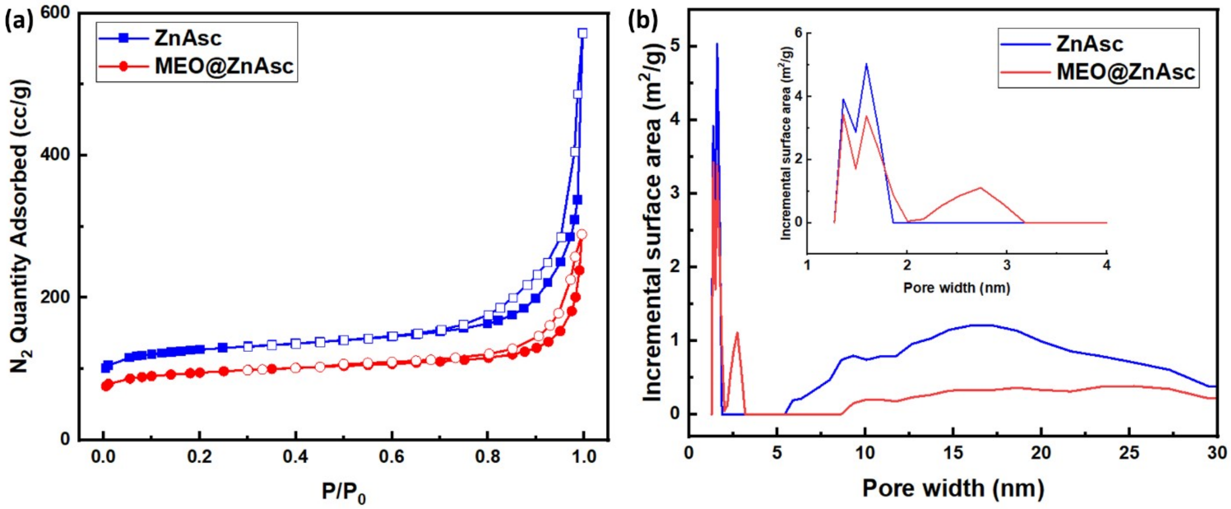

Figure S1. (a) N_2 sorption isotherms and (b) pore size distributions of ZnAsc and MEO@ZnAsc.

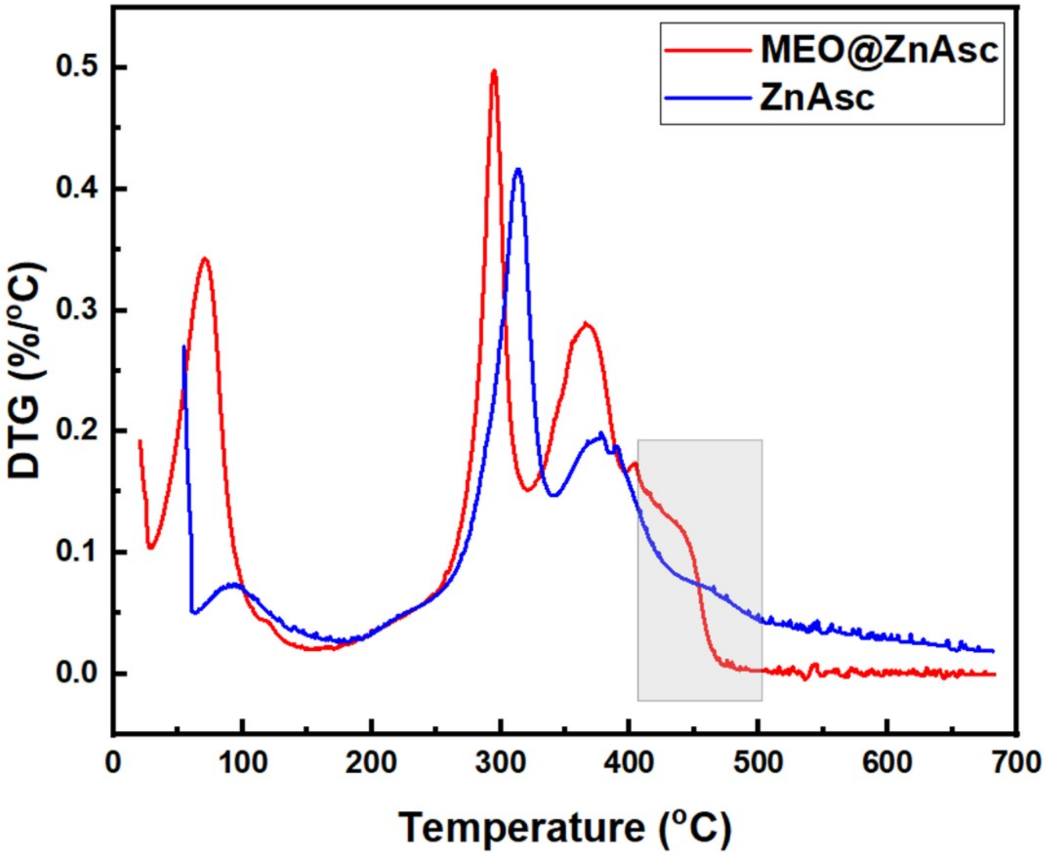

Figure S2. First derivative of the TGA profiles of ZnAsc and MEO@ZnAsc.

Table S2. Crystallite/particulate size determination by different methods (nm).

Method	ZnAsc	MEO@ZnAsc
**SEM**	20.9	23.3
**TEM**	18.6	18.1
**XRD**	16.5	15.6

Table S3. Antibacterial activities of Zn-based MOFs.

MOF	Inhibition zone diameter (mm)		Zn wt%	Ref.
	E. coli	*S. aureus*		
**Zn-MOF**	-	37.32	-	1
**GR-MOF-8**	15.96	20.20	29	2
**Ac MOF-5**	10	16	34	3
**Ac Zn-MOF**	14	16	26.4	3
**Ac TMU-3**	12	14	16.2	3
**IEF-24**	5.4	-	14.1	4
**Zn-MOF**	12.22	10.10	-	5
**Zn-MOF**	8.6	17	24.6	6
**[Zn(μ-4-hzba)$_2$]$_2$·4(H$_2$O)**	-	14.6	13.9	7
**ZnAsc**	**10.33**	**8**	**50**	**This work**

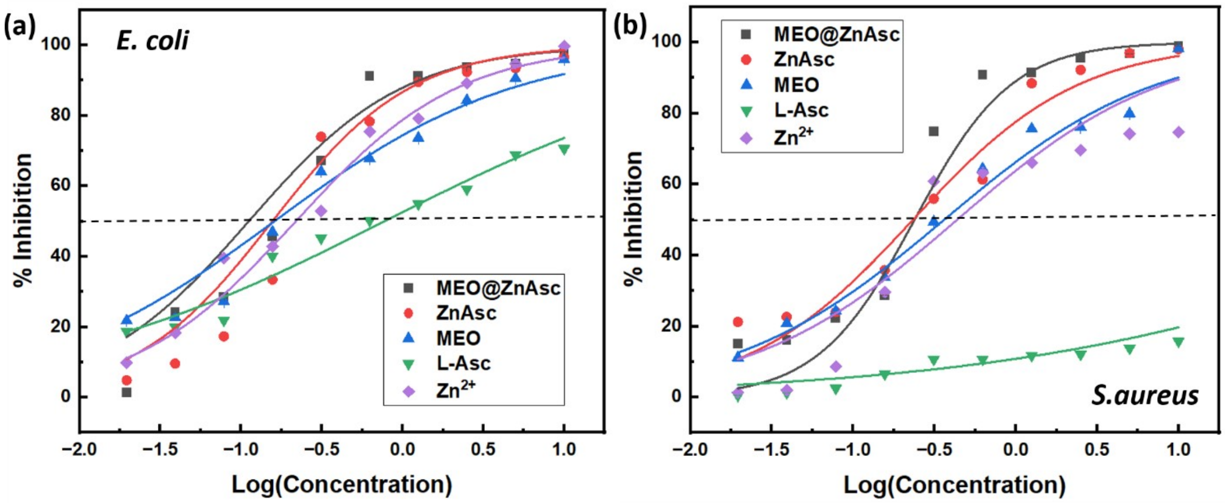

Figure S3. Dose response curves of the different samples against *E. coli* (a) and *S. aureus* (b) using broth microdilution method (dots represent experimental data and lines represent sigmoidal data fitting from which IC$_{50}$ values were calculated).

References

1. X. Xu, M. Ding, K. Liu, F. Lv, Y. Miao, Y. Liu, Y. Gong, Y. Huo and H. Li, *Front Chem*, 2023, **11**.
2. S. Rojas, A. García-García, T. Hidalgo, M. Rosales, D. Ruiz-Camino, P. Salcedo-Abraira, H. Montes-Andrés, D. Choquesillo-Lazarte, R. Rosal, P. Horcajada and A. Rodríguez-Diéguez, *Journal*, 2022, **12**.
3. M. Nakhaei, K. Akhbari, M. Kalati and A. Phuruangrat, *Inorganica Chimica Acta*, 2021, **522**, 120353.
4. S. N. Lelouche, L. Albentosa-González, P. Clemente-Casares, C. Biglione, A. Rodríguez-Diéguez, J. Tolosa Barrilero, J. C. García-Martínez and P. Horcajada, *Journal*, 2023, **13**.
5. C. Yuan, Y. Miao, Y. Chai, X. Zhang, X. Dong and Y. Zhao, *Journal*, 2023, **28**.
6. F. Akbarzadeh, M. Motaghi, N. P. S. Chauhan and G. Sargazi, *Heliyon*, 2020, **6**, e03231.
7. J. Restrepo, Z. Serroukh, J. Santiago-Morales, S. Aguado, P. Gómez-Sal, M. E. G. Mosquera and R. Rosal, *European Journal of Inorganic Chemistry*, 2017, **2017**, 574-580.
